# Awareness of hazards due to tobacco among people aged 15 years and older in Chongqing, China, in 2020: A cross-sectional analysis

**DOI:** 10.18332/tid/155933

**Published:** 2022-12-12

**Authors:** Qiu Chen, Jia Nan Dai, Xiao Dan Chen, Tian Qin, Wei Yun Lai, Yang Wang

**Affiliations:** 1 School of Public Health and Management, Chongqing Medical University, Chongqing, China; 2 Chongqing Health Education Institute, Chongqing, China; 3 Center for Lab Teaching and Management, Chongqing Medical University, Chongqing, China

**Keywords:** awareness rate, hazards due to tobacco, smoking, secondhand smoke

## Abstract

**INTRODUCTION:**

Tobacco smoke contains a large number of harmful substances and carcinogens. Smoking and secondhand smoke cause a variety of cancers and diseases, seriously endangering human health. However, the status and characteristics of the awareness of hazards due to tobacco among people aged ≥15 years in Chongqing, China, are still unknown.

**METHODS:**

A multistage stratified cluster random sampling method was used to select ten districts and counties in Chongqing Municipality, China and a total of 6622 people were investigated between August and October 2020. The chi-squared test was used to analyze the awareness of hazards due to tobacco in various populations after the data had been cleaned and weighted.

**RESULTS:**

In 2020, the awareness rates of people aged ≥15 years in Chongqing, China, about a specific disease caused by smoking were lung cancer (77.1%), heart disease (45.1%), stroke (40.1%), and penile erectile dysfunction (24.2%). However, only 22.1% of the respondents knew that smoking could simultaneously lead to all four diseases mentioned above. Adult lung cancer was the disease with the highest awareness rating (72.5%), followed by children’s lung disease (54.2%) and adult heart disease (46.1%). A total of 42.0% of respondents knew that secondhand smoke could cause the three diseases simultaneously. Only 22.0% of those correctly understood the harm of low-tar cigarettes. The logistic regression results showed that education level and occupation were risk factors for lack of awareness of hazards due to tobacco. In contrast, media campaigns on tobacco control were a protective factor.

**CONCLUSIONS:**

The awareness of hazards due to tobacco among people aged ≥15 years in Chongqing, China, still needs to be improved. More graphic health warning labels and mass media campaigns about the hazards of tobacco should be carried out to raise people’s awareness and warn about the health risks of smoking.

## INTRODUCTION

Previous studies have shown that smoking and secondhand smoke exposure lead to various respiratory diseases, cardiovascular diseases, and malignant tumors^1^. Globally, tobacco (smoking, secondhand exposure) remained a leading attributable risk factor for death in 2019, causing 8.71 million deaths (15.4% of all deaths in 2019)^2^. The direct economic burden associated with smoking in China in 2018 was US$82.63 billion, an increase of 93.0% from 2008^3^. The awareness of tobacco-related hazards (tobacco and secondhand smoke hazards) significantly impact smoking behaviour^4-7^. The primary consideration for quitting smoking is health issues; non-smokers are more aware of the risks due to tobacco than smokers^8^. Smokers’ awareness of risks due to tobacco was significantly associated with smokers’ willingness to quit smoking^9^. Therefore, the survey on awareness of hazards due to tobacco is significant for tobacco control, which can provide baseline data to evaluate the effectiveness of the current tobacco control measures and some guidance for future work in tobacco control. Our study aims to investigate demographic characteristics, awareness of hazards due to tobacco, and the affecting factors among adults aged ≥15 years in Chongqing, China, to provide practical measures to prevent and control tobacco epidemics.

## METHODS

### Study design

This cross-sectional study adopted a multistage stratified cluster random sampling design (stratified by rural vs urban) in each district between August and October 2020, after the COVID-19 epidemic was brought under control in Chongqing, China. Five urban and five rural communities were selected randomly. It was conducted by the Chongqing Health Education Institute in 8 districts (Wanzhou, Jiangbei, Beibei, Banan, Hechuan, Tongliang, Tongnan, Liangping) and 2 counties (Zhong, Fengjie). The sampling was divided into five stages. In the first stage, the Probability Proportionate to Size (PPS) sampling method was used to select the streets/towns, and three streets/towns were selected in each district (or county). In the second stage, the PPS method was used to select neighborhood committees/villages in each street/township. In the third stage, simple random sampling was used to determine areas, and the number of households in all neighborhood committees/administrative villages was required to be ≥750. If the number of households in the selected neighborhood committees/administrative villages was <750, the administrative villages/neighborhood committees were merged until all households met the requirements. If the number of households in the selected neighborhood committees/administrative villages was between 750 and 1500, the neighborhood committee/administrative village was used as the final sampling unit. If the selected neighborhood committee/administrative village had ≥1500 households, the neighborhood committee/administrative village needed to be divided into several subdistricts with roughly the same number of households (750–1500 in each subdistrict). A simple random sampling method was used for the final sampling unit. It was down to the professional technical institutions for tobacco control in each district and county to divide the subdistricts and report them to the Chongqing Health Education Institute. The Chongqing Health Education Institute was responsible for reviewing the subdistrict division and reporting it to the China CDC. The Tobacco Control Office of the China CDC was responsible for selecting the subdistricts and extracted a subdistrict among multiple subdistricts and divided by the drawing list. In the fourth stage, a random sampling method was adopted to select households, and 120 households were selected from each neighborhood committee/administrative village in the divided area. In the fifth stage, simple random sampling was adopted, and one member of the selected family aged ≥15 years was selected as the respondent. In total, 7200 respondents were included in the study.

### Participants

The participants were Chinese residents aged ≥15 years who lived in Chongqing at least one month before the survey, excluding people in collective living places such as student dormitories, military camps, prisons, or hospitals. All respondents signed an informed consent form. For minor participants aged between 15 and 18 years, consent for their participation was obtained from their parents or legal guardian as well as their own assent.

### Survey

The on-site survey adopted the form of a household survey. Investigators who had been trained professionally used tablet computers to conduct face-to-face inquiries with the respondents, and then the investigators filled in the survey results.

### Quality control

Another independent quality controller supervised and controlled the investigation process, kept a record of it, and randomly chose 10% of the questionnaires for review after the investigation was completed. A resampling investigation was needed if the response rate was below 85%.

### Definition of indicators

The adults in this study refer to people aged ≥15 years. Education level in this study only analyzed people aged ≥25 years, divided into four categories: primary school and lower; junior high school; high school; college and higher.

### Data management and transmission

After completing the daily survey, investigators at various monitoring sites in Chongqing were responsible for collecting and transmitting data, as well as uploading the data collected in the survey equipment to the designated server.

### Statistical analysis

SAS (SAS 9.4; SAS Institute, Cary, NC) software was used to clean the original data of the 2020 Chongqing Tobacco Survey. SPSS (SPSS V.21.0, Inc., Chicago, Illinois) software was used to perform essential weighting, non-response correction, and post-stratification correction on the cleaned data. Then, the complex sampling module was used for data analysis. Frequencies between groups were compared statistically using the Rao-Scott χ^2^ test. Logistic regression was used to analyze the factors affecting smoking attempts and behavior. Variables with statistical significance in the univariate logistic regression analysis were subjected to multivariate logistic regression analysis, including: age (25–44, 45–64, and ≥65 years); residence (urban, rural), whether or not had seen the media campaign for tobacco control publicity (yes/no); education level (elementary and lower, junior, senior, college and higher); occupation (government staff, service staff, teacher, medical staff, student, military, unemployed, retirees, or other); and whether or not there was secondhand smoke exposure (yes/no). An inspection level α=0.05 and a difference with p<0.05 were considered statistically significant.

## RESULTS

### Demographic characteristics

Of the 7200 respondents surveyed, 6622 completed individual questionnaires. After weighing the sample data, men accounted for 50.0%, and women accounted for 50.0%; urban residents accounted for 80.1%, and rural residents accounted for 19.2%; the composition ratio of the population in the age groups 25–44 and 45–64 years was relatively high, 32.1% and 37.1%, respectively; the composition ratio of the population with primary school and lower and junior high school education level was relatively high, 27.9% and 29.4%, respectively. Detailed demographic information is shown in Table 1.

**Table 1:** Demographic characteristics of tobacco hazard awareness among people aged ≥15 years in Chongqing, China, 2020 (N=6222)

*Demographic characteristics*	*Weighted*			*Unweighted sample size*
	*Rate (%)*	*95% CI*	*Sample size*	
**Total**	100	100	25710097	6622
**Region**				
Urban	80.1	56.5–92.6	20587620	4108
Rural	19.9	7.4–43.5	5122477	2514
**Gender**				
Male	50.0	46.7–53.3	12851132	3183
Female	50.0	46.7–53.3	12858965	3439
**Age** (years)				
15–24	13.9	7.1–25.2	3562632	182
25–44	32.1	27.3–37.3	8244576	1128
45–64	37.1	30.5–44.3	9546379	2741
≥65	16.9	13.8–20.6	4356510	2571
**Education level^a^**				
Elementary and lower	27.9	18.5–39.7	7146375	3404
Junior	29.4	23.6–35.9	7534938	1713
Senior	21.0	16.7–26.1	5395142	793
College and higher	21.7	11.9–36.4	5569952	702

a People aged ≥25 years.

### Awareness of hazards due to tobacco

The awareness rates for specific diseases caused by smoking were lung cancer (77.1%), heart disease (45.1%), stroke (40.1%), and penile erectile dysfunction (24.2%). However, only 22.1% of the respondents knew that smoking could simultaneously lead to all four diseases mentioned above. No significant difference was found between urban and rural populations in the awareness rate of stroke, heart disease, and PED caused by smoking (p>0.05). The awareness rate of lung cancer caused by smoking was significantly different between urban (81.9%) and rural populations (59.0%) (p<0.05). Although both urban and rural populations knew that smoking caused all the above diseases, the difference was not significant (p>0.05). There was no significant difference between the sexes in smoking causing one or all of the above diseases (p>0.05). With increasing age, the awareness rates of one or all smoking-induced diseases decreased, and the difference was statistically significant (p<0.001). No significant difference was found in the awareness rate of smoking-induced stroke among the different age groups (p>0.05). The awareness rate of one or more of the above smoking-related diseases increased with higher levels of education. This difference was statistically significant (p<0.001). The awareness rate of different diseases is shown in Table 2.

**Table 2:** The awareness rate (%) of smoking-related diseases, secondhand smoke-related diseases, and understanding the hazards due to low-tar cigarettes among people aged ≥15 years in Chongqing, China, 2020 (N=6222)

*Variable*	*Region*		*p*	*Gender*		*p*	*Age (years)*				*p*	*Education level^a^*				*p*
	*Urban*	*Rural*		*Male*	*Female*		*15–24*	*25–44*	*45–64*	*≥65*		*E*	*J*	*S*	*C*	
**Awareness rate of smoking-related diseases**																
Stroke	40.8	37.6	0.726	40.8	39.5	0.637	49.8	47.7	35.1	28.9	0.091	23.7	35.2	47.0	62.5	<0.001*
Heart disease	46.6	39.1	0.365	45.3	45.0	0.849	59.7	53.1	39.1	31.2	0.029*	25.0	40.5	53.1	68.0	<0.001*
Lung cancer	81.9	59.0	0.013*	77.0	77.2	0.292	96.3	84.6	72.1	58.1	<0.001*	55.6	75.8	85.2	91.9	<0.001*
Erectile dysfunction	26.4	15.4	0.056	24.4	24.0	0.640	34.8	31.1	19.1	13.2	<0.001*	11.2	15.8	26.8	49.9	<0.001*
All above diseases	23.9	14.9	0.112	21.9	22.3	0.596	29.1	28.5	17.9	12.8	0.003*	10.4	14.4	25.6	46.1	<0.001*
**Awareness rate of diseases due to secondhand smoke**																
Heart disease in adults	47.8	39.3	0.343	45.2	47.0	0.532	65.4	54.8	39.4	28.5	0.003*	23.4	40.8	56.2	68.4	<0.001*
Lung disease in children	56.5	45.1	0.185	52.2	56.2	0.101	74.4	64.1	47.5	33.7	0.005*	27.7	51.7	66.6	75.1	<0.001*
Lung disease in adults	77.7	51.7	0.003*	72.4	72.6	0.578	92.0	81.9	66.8	51.2	<0.001*	47.1	72.8	81.1	90.1	<0.001*
All above diseases	43.4	36.2	0.288	41.0	43.0	0.439	59.6	51.2	35.6	23.9	0.01*	20.1	36.5	53.3	63.2	<0.001*
**Understanding the hazards due to low-tar cigarettes**																
Correct awareness	26.0	6.0	0.007*	22.7	21.3	0.661	33.5	27.6	17.1	12.8	0.004*	9.6	16.6	22.7	42.7	<0.001*

a E: Elementary and lower. J: Junior. S: Senior. C: College and higher.

* p<0.05

### Awareness of hazards due to secondhand smoke

The awareness rates of the specific diseases caused by secondhand smoke among respondents were adult lung cancer (72.5%), childhood lung disease (54.2%), and adult heart disease (46.1%). Only 42.0% knew that secondhand smoke could cause all the above diseases. There was no significant difference in the awareness rate of secondhand smoke causing adult heart disease and childhood lung disease between urban and rural populations (p>0.05). The urban population (77.7%) was more aware of adult lung cancer caused by secondhand smoke than the rural population (51.7%). The difference was significant (p<0.001). No significant difference was found in the awareness rate of secondhand smoke causing all the above diseases between urban and rural populations (p>0.05). There was no significant difference in the awareness rate of secondhand smoke causing one or all of the above diseases between the sexes (p>0.05). With increasing age, the awareness rate of secondhand smoke causing one or all of the above diseases decreased, and the difference was statistically significant (p<0.05). With the increased education level, the awareness rate of one or all diseases caused by secondhand smoke increased, and the difference was statistically significant (p<0.001). Details of the information are shown in Table 2.

### Awareness of hazards due to low-tar cigarettes

Only 22.0% of the respondents correctly understood that the hazards due to low-tar cigarettes were not different from those of ordinary cigarettes; 22.3% had incorrect perceptions and 55.7% did not know about the hazards due to low-tar cigarettes. The correct awareness of the hazards due to low-tar cigarettes among the urban population (26.0%) was significantly higher than that among the rural population (6.0%), and the difference was statistically significant (p<0.001). Men (22.7%) were slightly more aware of the hazards due to low-tar cigarettes than women (14.5%), but the difference was not statistically significant (p>0.05). With increasing age, the correct awareness rate of hazards due to low-tar cigarettes decreased, and the difference was statistically significant (p<0.001). With a higher education level, the correct awareness rate of hazards due to low-tar cigarettes increased, and the difference was statistically significant (p<0.001). A total of 42.7% of the population with a junior college degree and higher had a correct awareness of hazards due to low-tar cigarettes, and only 9.6% of those with a primary degree and lower had a correct awareness of hazards due to low-tar cigarettes (Table 2).

### Multivariate analysis of awareness of hazards due to tobacco

All variables were studied in univariate analysis; those with p<0.05 were included in multivariate logistic regression analysis, including age (25–44, 45–64, and ≥65 years); residence (urban, rural), whether or not had seen the media campaign for tobacco control publicity (yes/no); education level (elementary and lower, junior, senior, college and higher); occupation (government staff, services staff, teacher, medical staff, student, military, unemployed, retirees, and other); and whether or not there was secondhand smoke exposure (yes/no) (Table 3). The results showed that education level (high school degree or lower) was a risk factor for the lack of awareness of hazards due to tobacco, while media campaigns on tobacco control were protective factors (Table 4). Furthermore, the education level (high school and lower) and occupation (agriculture, forestry, animal husbandry, fisheries, and services staff) were risk factors for lack of awareness of diseases caused by secondhand smoke, while media campaigns on tobacco control and living in the cities were protective factors (Table 4). Additionally, education level (high school and lower) was a risk factor for lack of awareness of hazards due to low-tar cigarettes, while media campaigns on tobacco control and living in the cities were protective factors (Table 4).

**Table 3:** Univariate analysis results of tobacco hazard awareness among people aged ≥15 years in Chongqing, China, 2020 (N=6222)

*Characteristics*	*All disease – A^b^ OR (95% CI)*	*All disease – B^c^ OR (95% CI)*	*Correct awareness of low-tar cigarettes^d^ OR (95% CI)*
**Total**	22.1 (14.7–31.7)	42.0 (34.0–50.4)	22.0 (9.4–43.4)
**Region**			
Urban	23.9 (15.9–34.3)	43.4 (34.7–52.6)	26.0 (11.7–48.3)
Rural	14.9 (7.8–26.7)	36.2 (25.0–49.1)	6.0 (3.0–11.6)
p	0.119	0.288	0.007*
**Gender**			
Male	21.9 (14.6–31.3)	41.0 (32.8–49.8)	22.7 (8.3–48.9)
Female	22.3 (14.7–32.3)	43.0 (34.4–51.9)	21.3 (10.6–38.2)
p	0.601	0.434	0.661
**Age** (years)			
15–24	29.1 (19.7–41.7)	59.6 (42.1–75.0)	33.5 (13.1–62.9)
25–44	28.5 (19.5–39.6)	51.2 (43.5–58.9)	27.6 (12.5–50.4)
45–64	17.9 (11.7–26.4)	35.6 (26.2–46.4)	17.1 (7.8–33.3)
≥65	12.8 (7.0–22.2)	23.9 (14.6–36.5)	12.8 (5.8–25.9)
p	0.002*	0.01*	0.004*
**Education level^a^**			
Elementary and lower	10.4 (5.3–19.4)	20.1 (12.4–31.0)	9.6 (5.3–16.9)
Junior	14.4 (9.2–21.8)	36.5 (29.1–44.6)	16.6 (7.4–32.8)
Senior	25.6 (17.6–35.6)	53.3 (44.5–62.0)	22.7 (11.2–40.5)
College and higher	46.1 (37.8–54.6)	63.2 (57.1–68.9)	42.7 (24.0–63.8)
p	<0.001*	<0.001*	<0.001*
**Occupation**			
Agriculture, forestry, animal husbandry, and fisheries	10.4 (4.9–20.8)	24.8 (14.2–39.7)	8.3 (3.7–17.6)
Government staff	49.9 (25.5–74.1)	66.2 (46.8–81.4)	38.5 (13.3–71.9)
Services staff	25.2 (17.3–35.2)	48.7 (39.8–57.7)	36.1 (14.9–64.7)
Teacher	¶	55.8 (41.6–69.0)	¶
Medical staff	54.9 (29.4–78.1)	77.5 (56.6–90.1)	¶
Student	¶	48.2 (22.2–75.3)	¶
Military	¶	¶	¶
Unemployed	16.3 (8.3–29.8)	37.4 (28.8–46.8)	15.9 (8.0–28.9)
Retirees	20.0 (14.4–27.0)	41.9 (34.4–49.8)	22.7 (12.0–38.7)
Other	28.0 (20.2–37.4)	50.8 (41.5–60.1)	18.2 (10.7–29.3)
p	0.003*	0.001*	0.007*
**Smoking status**			
Smoker	19.9 (11.3–32.7)	42.1 (32.7–52.0)	23.3 (8.0–51.7)
Non-smoker	22.8 (15.4–32.3)	41.39 (33.7–50.7)	21.6 (9.9–40.9)
p	0.370	0.965	0.616
**Seen tobacco control publicity?**			
Yes	29.3 (21.0–39.2)	56.3 (48.5–63.8)	25.6 (11.4–47.8)
No	13.3 (7.8–21.6)	25.4 (18.7–33.4)	17.9 (7.2–37.9)
p	<0.001*	<0.001*	0.005*
**Secondhand smoke exposure?**			
Yes	26.4 (18.2–36.6)	46.6 (37.3–56.2)	27.4 (12.3–50.4)
No	17.5 (11.6–25.5)	35.6 (28.4–43.4)	14.8 (8.0–25.6)
p	0.017*	0.004*	0.024*

a People aged ≥25 years.

b Includes stroke, heart disease, lung cancer and erectile dysfunction.

c Includes adult heart disease, childhood lung disease, and adult lung cancer.

d Correct awareness rate of low-tar cigarettes.

* p<0.05.

¶ Refers to <25 people.

**Table 4:** Multivariate analysis results of awareness of hazards due to tobacco, secondhand smoke, and low-tar cigarettes among people aged ≥15 years in Chongqing, China, 2020 (N=6222)

*Variables^a^*	*Multivariate-adjusted*	
	*AOR (95% CI)^b^*	*p*
**Awareness of hazards due to tobacco**		
**Intercept**	1.869 (0.86–4.065)	0.100
**Age** (years)		
25–44	1.273 (0.767–2.112)	0.304
45–64	1.002 (0.685–1.466)	0.989
≥65 (Ref.)	1	
**Education level**		
Elementary and lower	5.311 (2.765–10.201)	0.000*
Junior	4.347 (2.999–6.301)	0.000*
Senior	2.498 (1.622–3.847)	0.001*
College and higher (Ref.)	1	
**Seen anti-tobacco media campaign**		
Yes	0.419 (0.317–0.555)	0.000*
No (Ref.)	1	
**Secondhand smoke exposure**		
Yes	0.697 (0.412–1.179)	0.152
No (Ref.)	1	
**Occupation**		
Agriculture, forestry, animal husbandry, and fisheries	2.01 (0.843–4.796)	0.101
Government staff	0.869 (0.492–1.535)	0.585
Services staff	1.169 (0.643–2.126)	0.564
Medical staff	0.285 (0.054–1.493)	0.119
Unemployed	1.454 (0.647–3.266)	0.317
Retirees	1.473 (0.79–2.746)	0.189
Other (Ref.)	1	
**Awareness of hazards due to secondhand smoke**		
**Intercept**	1.028 (0.607–1.743)	0.907
**Age** (years)		
25–44	0.692 (0.405–1.182)	0.152
45–64	0.796 (0.574–1.105)	0.148
≥65 (Ref.)	1	
**Education level**		
Elementary and lower	5.325 (3.364–8.43)	0.000*
Junior	2.974 (2.004–4.412)	0.000*
Senior	1.534 (1.099–2.141)	0.018*
College and higher (Ref.)	1	
**Seen anti-tobacco media campaign**		
Yes	0.306 (0.221–0.424)	0.000*
No (Ref.)	1	
**Secondhand smoke exposure**		
Yes	0.791 (0.582–1.074)	0.115
No (Ref.)	1	
**Occupation**		
Agriculture, forestry, animal husbandry, and fisheries	2.228 (1.225–4.052)	0.015
Government staff	1.318 (0.601–2.887)	0.441
Services staff	2.332 (1.358–4.005)	0.007
Teachers	2.726 (0.773–9.616)	0.104
Medical staff	0.894 (0.247–3.243)	0.847
Students	0.000	0.000
Unemployed	1.256 (0.877–1.799)	0.182
Other (Ref.)	1	
**Awareness of hazards due to low-tar cigarettes**		
**Intercept**	15.794 (3.524–70.797)	0.003
**Age** (years)		
25–44	0.699 (0.459–1.064)	0.085
45–64	0.915 (0.706–1.186)	0.453
≥65 (Ref.)	1	
**Education level**		
Elementary and below	3.242 (2.271–4.627)	0.000
Junior	2.771 (2.05–3.747)	0.000
Senior	2.525 (1.786–3.568)	0.000
College and above (Ref.)	1	
**Seen anti-tobacco media campaign**		
Yes	0.582 (0.415–0.816)	0.006
No (Ref.)		
**Region**		
City	0.236 (0.059–0.948)	0.044
Rural (Ref.)	1	
**Occupation**		
Agriculture, forestry, animal husbandry, and fisheries	1.18 (0.308–4.525)	0.783
Government staff	0.853 (0.287–2.535)	0.744
Services staff	0.674 (0.313–1.452)	0.270
Unemployed	1.335 (0.699–2.551)	0.333
Retirees	0.701 (0.374–1.313)	0.228
Other (Ref.)	1	
**Secondhand smoke exposure**		
Yes	0.641 (0.385–1.067)	0.079
No (Ref.)	1	

a All predictors in Table (including age, education level, occupation, whether the participants saw the media campaign for tobacco control publicity, place of residence, and whether there was secondhand smoke exposure).

b The AOR (adjusted odds ratio) with Ref. (reference category) is written so that the survey respondents could select only one choice and not select all that are applicable. The reference category in the latter variables means not having that factor.

* p<0.05.

## DISCUSSION

Among the leading causes of preventable morbidity and mortality worldwide, smoking and secondhand smoke exposure remain threats to public health^10^. In recent years, significant developments have been made in media campaigns, but the lack of awareness of hazards due to tobacco remains a challenge. Awareness about risks due to tobacco could form the foundation for promoting positive beliefs and attitudes about tobacco exposure. A study of smoking hazard awareness revealed that an incorrect awareness of hazards due to smoking increases the likelihood of smoking^11^. Yang et al.^12^ found that awareness of the health hazards due to tobacco is still relatively poor in China, particularly among rural residents and people with low education level. They suggested that the current health warnings on the cigarette package should be revised to a great extent. A study from Tianjin, China, highlighted that smoking data from a system of death certification could be used to evaluate the effects of tobacco control measures^13^.

Our results show that the awareness of hazards due to smoking among people in Chongqing, China, was <50%, except for lung cancer. Only 24.2% of the respondents knew that smoking might contribute to penile erectile dysfunction (PED). Verze et al.^14^ found that people have a lower awareness rate of PED caused by smoking and secondhand smoking. Similar conclusions were drawn by Millett et al.^15^. Smoking is widely recognized as the leading risk factor for developing PED. Gades et al.^16^ demonstrated that men who smoked at any time had a greater likelihood of PED and showed a dose-response correlation with the number of years of smoking and cigarettes smoked. A systematic review by Carreras et al.^17^ showed that smoking is a significant risk factor for PED in young men. The impact of smoking on PED depends on the dose; the higher the dose, the more severe the PED, a condition that appears irreversible after smoking cessation. Additionally, our results indicate that the awareness of cardiovascular diseases (CVDs) caused by smoking and secondhand smoke was <50%. A Bayesian hierarchical model was used by Lee et al.^18^ to evaluate the relationships between smoking and cardiovascular diseases. They concluded that smoking and passive smoking were significantly associated with CVD. A significant dose-response relationship was found by Markidan et al.^19^ between daily cigarette use and ischemic stroke.

Only 22.1% of respondents knew that smoking could simultaneously cause stroke, heart disease, lung cancer, and erectile dysfunction. Most survey respondents knew that smoking causes lung cancer, but awareness of the other hazards due to smoking was low. In addition, our study revealed that only a small portion of the respondents understood the hazards of low-tar cigarettes. Falcone et al.^20^ also found low awareness of ‘light’ cigarettes in their study. Notably, the awareness rate of hazards due to low-tar cigarettes is very low in rural residents and people with elementary school education and below, at 6.0% and 9.6%, respectively. Gan et al.^21^ found that urinary cotinine, trans-3’-hydroxycotinine, and polycyclic aromatic hydrocarbons (PAHs) in low-tar cigarette smokers were not related to tar release from cigarettes, indicating that low-tar cigarettes do not deliver lower doses of nicotine and carcinogens than regular cigarettes. In addition, Lee et al.^22^ reported that low/ultralow tar and nicotine cigarettes cause smokers to change their smoking patterns to compensate for low tar or nicotine levels, leading to the same exposure level of tar as the regular cigarette. It is suggested that the labeling, advertising, and branding of all tobacco products with low tar or light claims be carefully regulated under a ‘not false or misleading’ standard. The multivariate logistic regression analysis suggested that high school and lower education were risk factors for lack of awareness of hazards due to tobacco. Furthermore, occupation in agriculture, forestry, animal husbandry, and fisheries were risk factors for lack of awareness of hazards due to tobacco, while tobacco control media campaigns were protective factors. According to Wang et al.^23^, occupation and education level were significantly associated with smoking behaviors, with higher education level being associated with fewer smoking behaviors. However, further research should evaluate the observed relationship.

In terms of the results on the awareness of hazards due to tobacco among the Chongqing population, the publicity of tobacco-related diseases other than lung cancer should be strengthened, especially of PED; moreover, efforts should be made to let people know that hazards due to low-tar cigarettes are no different from those of regular tobacco. Only by making the population aware of the great hazards due to tobacco, can the idea of smoking be discouraged and the awareness of smoking cessation be awakened. The World Health Organization released the MPOWER tobacco control strategy in 2008 (Monitoring tobacco use and prevention policies; Protecting people from tobacco smoke; Offering help to quit tobacco use; Warning about the dangers of tobacco; Enforcing bans on tobacco advertising, promotion, and sponsorship; and Raising taxes on tobacco)^24^. In recent years, it has been reported that smoking and secondhand smoke exposure are strongly associated with sociodemographic characteristics^25^. To the best of the authors’ knowledge, there is currently insufficient evidence about awareness of hazards due to tobacco. Therefore, studies of demographic characteristics and awareness of risks due to tobacco are important not only to evaluate the effectiveness of tobacco control measures but also provide guidance for future tobacco control efforts.

Media campaigns are critical for enhancing public awareness of hazards due to tobacco and should be strengthened in the future to support these people in achieving and sustaining effective self-management. In addition to conventional media campaigns such as television, radio, billboards, bulletin boards, and brochures, and some targeted tobacco control strategies such as a more comprehensive cigarette packaging design and warning of erectile dysfunction, could be adopted. Furthermore, hazards due to tobacco monitoring should also be conducted on an ongoing basis to evaluate the effectiveness of hazards due to tobacco publicity. Tobacco control efforts can be strengthened through targeted interventions to enhance the awareness of hazards due to tobacco among populations with specific occupations and low education level.

### Limitations

This study has some limitations. First, the presented awareness of hazards due to tobacco only reflects the situation of the population in Chongqing, China. Second, the survey used a closed questionnaire, making it difficult to obtain more detailed and in-depth information. Third, the survey is affected by household surveys. Some respondents had concerns about privacy and personal information and may have been unwilling to give true answers, resulting in information bias. Fourth, the hazards due to tobacco are extensive and deep, and the survey only uses the above tobacco-related diseases as evaluation indicators. Last, the present study did not consider assessing harm awareness of novel generation tobacco and nicotine products, such as e-cigarettes and heated tobacco products, which are currently of great interest. Further research on the understanding of hazards due to e-cigarettes is warranted.

## CONCLUSIONS

This survey shows that people aged ≥15 in Chongqing, China, have a significantly less comprehensive awareness of the tobacco smoke, and only a small portion of people correctly understand the hazards of low-tar cigarettes. Local health education departments are called upon to increase the awareness of risks due to tobacco campaigns or activities (e.g. issuing health brochures in conjunction with hospital smoking cessation clinics) to raise comprehensive awareness of hazards due to tobacco among people aged ≥15 years, as part of the national tobacco control strategy.

## Data Availability

The data supporting this research are available from the authors on reasonable request.

## References

[1] Wang C, Xiao D (2021). 2020 Report on Health Hazards of Smoking in China: an Updated Summary. In Chinese. Chinese Circulation Journal.

[2] GBD 2019 Risk Factors Collaborators (2020). Global burden of 87 risk factors in 204 countries and territories, 1990-2019: a systematic analysis for the Global Burden of Disease Study 2019. Lancet.

[3] Zhang Y, Wu S (2021). Analysis on direct economic burden of diseases attributable to smoking among Chinese residents. Chinese Journal of Hospital Statistics.

[4] Aswathy S, Syama S, Georgy S (2021). Tobacco use and exposure to second-hand smoke among high school students in Ernakulum district, Kerala: A cross-sectional study. Public Health Pract (Oxf).

[5] Yang X, Chen W, Peng X (2020). The prevalence of smoking in male medical students from 16 Chinese medical colleges and the associated factors. Ann Palliat Med.

[6] Szymański J, Ostrowska A, Pinkas J, Giermaziak W, Krzych-Fałta E, Jankowski M (2022). Awareness of Tobacco-Related Diseases among Adults in Poland: A 2022 Nationwide Cross-Sectional Survey. Int J Environ Res Public Health.

[7] Míguez Varela MC, Pereira B (2018). Prevalence and Risk Factors Associated with Smoking in Early Pregnancy. Prevalencia y factores de riesgo del consumo de tabaco en el embarazo temprano. Rev Esp Salud Publica.

[8] Jiang B, Ma AJ, Xie C (2020). Study on intention of smoking concession, awareness of smoking hazards and impact on smoking status in residents aged 18-65 years in Beijing. In Chinese. Zhonghua Liu Xing Bing Xue Za Zhi.

[9] Akiyama O, Nakamura M, Tabuchi T (2018). Awareness of harm to others from secondhand smoke and smokers’ interest in smoking cessation. In Japanese. Nihon Koshu Eisei Zasshi.

[10] Koronaiou K, Al-Lawati JA, Sayed M (2021). Economic cost of smoking and secondhand smoke exposure in the Gulf Cooperation Council countries. Tob Control.

[11] Kang LJ, Kim HS (2005). Risk and Protective Factors Related to Cigarette Smoking among Korean Male High School Students. In Korean. Consumption Cult Study.

[12] Yang Y, Wang JJ, Wang CX, Li Q, Yang GH (2010). Awareness of tobacco-related health hazards among adults in China. Biomed Environ Sci.

[13] Li W, Xue X, Li D (2022). Attributable fraction of tobacco smoking on selected cancer deaths in the past decade using mortality case-control study in Tianjin, China. Tob Induc Dis.

[14] Verze P, Margreiter M, Esposito K, Montorsi P, Mulhall J (2015). The Link Between Cigarette Smoking and Erectile Dysfunction: A Systematic Review. Eur Urol Focus.

[15] Millett C, Wen LM, Rissel C (2006). Smoking and erectile dysfunction: findings from a representative sample of Australian men. Tob Control.

[16] Gades NM, Nehra A, Jacobson DJ (2005). Association between smoking and erectile dysfunction: a population-based study. Am J Epidemiol.

[17] Carreras G, Lugo A, Gallus S (2019). Burden of disease attributable to second-hand smoke exposure: A systematic review. Prev Med.

[18] Lee W, Hwang SH, Choi H, Kim H (2017). The association between smoking or passive smoking and cardiovascular diseases using a Bayesian hierarchical model: based on the 2008-2013 Korea Community Health Survey. Epidemiol Health.

[19] Markidan J, Cole JW, Cronin CA (2018). Smoking and Risk of Ischemic Stroke in Young Men. Stroke.

[20] Falcone M, Bansal-Travers M, Sanborn PM, Tang KZ, Strasser AA (2015). Awareness of FDA-mandated cigarette packaging changes among smokers of ‘light’ cigarettes. Health Educ Res.

[21] Gan Q, Lu W, Xu J (2010). Chinese ‘low-tar’ cigarettes do not deliver lower levels of nicotine and carcinogens. Tob Control.

[22] Lee PN, Fry JS, Hamling JS (2017). Investigation into the risk of ultra-low tar cigarettes and lung cancer. Regul Toxicol Pharmacol.

[23] Wang Q, Shen JJ, Sotero M, Li CA, Hou Z (2018). Income, occupation and education: Are they related to smoking behaviors in China?. PLoS One.

[24] World Health Organization (2008). MPOWER: A Policy Package to Reverse the Tobacco Epidemic.

[25] Zheng Y, Ji Y, Dong H, Chang C (2018). The prevalence of smoking, second-hand smoke exposure, and knowledge of the health hazards of smoking among internal migrants in 12 provinces in China: a cross-sectional analysis. BMC Public Health.

